# The impact of gold mining and agricultural concessions on the tree cover and local communities in northern Myanmar

**DOI:** 10.1038/srep46594

**Published:** 2017-04-24

**Authors:** Sarah Papworth, Madhu Rao, Myint Myint Oo, Kyaw Thinn Latt, Robert Tizard, Thomas Pienkowski, L. Roman Carrasco

**Affiliations:** 1 School of Biological Sciences, Royal Holloway University of London, United Kingdom; 2 Department of Biological Sciences, National University of Singapore, Singapore; 3 Wildlife Conservation Society Singapore, Singapore; 4 Wildlife Conservation Society Myanmar, Yangon, Myanmar

## Abstract

Myanmar offers unique opportunities for both biodiversity conservation and foreign direct investment due to projected economic growth linked to natural resource exploitation. Industrial-scale development introduces new land uses into the landscape, with unknown repercussions for local communities and biodiversity conservation. We use participatory mapping of 31 communities, focus groups in 28 communities, and analyses of forest cover change during 2000–2010 using MODIS vegetation continuous fields images, to understand the social and environmental impacts of gold mining and agricultural concessions in Myanmar’s Hukaung Valley (~21,800 km^2^). Local communities, particularly the poorest households, benefit from work and trade opportunities offered by gold mining and agricultural companies but continue to depend on forests for house construction materials, food, and income from the sale of forest resources. However, gold mining and agricultural concessions reduce tree cover, potentially reducing access to forest resources and further marginalizing these households. Our analyses do not provide evidence that long-term resident communities contributed to forest cover loss between 2000 and 2010. We argue that landscape management, which recognizes local community rights to customary community use areas, and appropriate zoning for commercial land uses and protected areas could contribute to both local livelihoods and protect biodiversity throughout Myanmar during economic growth.

Following the lifting of trade embargos since 2011, international economic interests can investigate opportunities offered by Myanmar’s relatively abundant natural resources and human capital^1^,^2^. Myanmar’s annual economic growth rate was 8.5% in 2014 and was projected to grow at a little over 8% per annum until 2017^3^. This economic growth is tightly linked with natural resource exploitation – including rapid development in the energy, infrastructure and agriculture sectors, and extractive industries^4^,^5^. Globally, these same sectors have been closely associated with local biodiversity losses and deforestation^6^,^7^,^8^,^9^,^10^,^11^ and protected area downgrading, downsizing, and degazettement^12^, but will offer opportunities for employment and economic growth in Myanmar. As Myanmar has some of the largest areas of remaining natural forest in Southeast Asia^13^ and a large rural population^14^, assessing the potential impacts of novel land use related to natural resource extraction is particularly important. Ambiguity over access rights has meant the traditional use areas of some communities in Myanmar have been granted to external groups for industrial-scale commercial land uses such as agricultural or mining development^15^. When this happens, the impact of these land uses on both communities and the natural environment are not well understood – in addition to the direct impact of new land use types and potential immigration, traditional land uses may be displaced to new areas^16^.

One example of an industrial-scale commercial land use which may expand in Myanmar is mining. Myanmar has diverse mineral resources, including gold^5^,^17^. The price of gold has more than quadrupled since 2001^18^, leading to increased rates of global extraction^19^. Even small-scale, artisanal gold mining can have negative environmental and social impacts such as deforestation^7^,^20^, water contamination^21^, mass immigration and even the death of workers from metal poisoning^22^. Yet gold mining is also an important source of revenue for local communities^23^ and governments^24^, and there are methods for reducing, and even reversing, the negative impacts of gold mining^6^,^25^. Although the impact of gold mining on local environmental quality is largely negative^6^,^7^, the impacts on local communities are less clear-cut, with communities experiencing both costs (e.g. increased exposure to heavy metals) and benefits (e.g. increased income and livelihood opportunities) as mining expands^23^,^26^,^27^.

Like gold mining, the environmental and social impacts of biofuel concessions are hotly debated, even as biofuels are heralded as a sustainable alternative to fossil fuels^28^. Although biofuel production can yield positive results for local communities, these benefits may be distributed unequally and observed implications of biofuel development on the local environment and livelihoods suggest complex interactions and responses^28^,^29^,^30^. Furthermore, in spite of the potential of biofuels to reduce fossil fuel use, the local environment where biofuels are grown can suffer from deforestation and water and air pollution^30^ and global food security may be negatively impacted if agricultural lands are devoted to biofuel production^31^. Although expectations of biofuel’s potential in South East Asia have not been realized^32^, various agricultural concessions for growing biofuels have been granted in Myanmar^33^, and expansion of industrial agriculture in Asia has led to widespread social and environmental change. Agricultural development has and will continue to underpin poverty alleviation across Asia^34^,^35^, yet the benefits of agricultural expansion varies across groups and scales. For example, Schneider reports at least 27 forced evictions, driven by industrial agricultural expansion, affecting an estimated 23,000 people in Cambodia in 2009^36^. The impacts of industrial agriculture on forest cover and biodiversity are significant. An estimated 15.1 million hectares of tropical forest were lost in southeast Asia between 2000 and 2010, principally driven by agricultural expansion^37^. These forests include globally important biodiversity hotspots and their loss is a major driver of species extinction^38^,^39^,^40^.

This study focuses on the social and environmental impacts of gold mining and agricultural concessions in the Hukaung Valley in northern Myanmar. With Myanmar’s increased market access and a growing economy based on natural resource exploitation—industrial-scale extractive industries such as mining, logging and agricultural concessions^4^ –understanding the impacts of these industries on the local environment and communities is timely. We focus here on the Hukaung Valley in northern Myanmar, which covers approximately 21,800 km^2^ and has already experienced growth in industrial-scale natural resource extraction due to increases in gold mining and the granting of land for agricultural concessions. The interaction between the development of these concessions, gold mining and natural resource use by communities represents a unique landscape that grants the opportunity to understand potential environmental and social impacts of industrial-scale land use and draw lessons for future landscape management in Myanmar. Understanding the complex interactions and feedbacks between socio-economic and ecological systems that emerge through the growth of gold mines and creation of agricultural concessions can be used to propose policies that reconcile economic development and biodiversity conservation in Myanmar.

Mining in Myanmar is nominally the responsibility of the Ministry of Mines^41^, though gold mines in the Kachin region where Hukaung Valley is located have been controlled and used by the Kachin Independence Organisation as a source of income^42^. Local communities have mined gold since before the early 20^th^ century, but gold mining in the Hukaung Valley landscape has substantially increased since the mid-1990s after a ceasefire between the government and Kachin ethnic forces, and by 2001 the nearby Hpakant area was flooded by an estimated half a million migrants from other areas of Myanmar^43^. Since then, mining has further expanded, with Kachin state containing 40% of all new mining areas developed in Myanmar between 2002 and 2015^17^. The impacts of this increase in mining, which represents one of numerous threats to the environment and communities, has not yet been quantified in the Hukaung Valley. Two agricultural concessions, Jadeland and Yuzana, each of 40.47 km^2^, were granted in Hukaung Valley in 2006. Although the Jadeland concession remains largely undeveloped, the main crops grown in Yuzana have been jatropha (*Jatropha spp*.) and cassava (*Manihot esculenta*), intended for use as biofuels.

Gold mining and agricultural concessions have expanded in Hukaung Valley, but the environmental and social impacts of this expansion for local communities have not been quantified. There are 64 communities in the Hukaung Valley (some founded as early as the 16^th^ century), where the main livelihood activities are agriculture (livestock, rice paddies and shifting cultivation) and hunting^44^, although some individuals collect and sell natural resources or own small businesses. Communities in the Hukaung Valley are ethnically heterogeneous and there are significant cultural differences between communities of the dominant ethnicities of Kachin and Naga. There are also households from minority Bamar, Chin, Lesu, Shan and Rakhine groups. Households also grow opium to sell to miners, which has been suggested as a significant driver of forest loss along river banks in the Hukaung Valley landscape^45^. Opium use in mining areas in Hukaung Valley is widespread as it allows miners to work longer hours^43^, thus opium cultivation could grow with gold mining. In addition to gold mining, amber mining is also conducted, largely at a single location within the valley^46^ and there are explorative expeditions for gas and oil.

We combine quantitative analysis of satellite images and qualitative analysis of rapid rural appraisals to understand the impact of gold mining and agricultural concessions on the environment and communities in Myanmar’s Hukaung Valley. Specifically, this research asked:

(1) Which areas of the local environment are used by communities, which natural resources do they value, and how is availability of these resources perceived to have changed as a result of expanding gold mining and agricultural concessions?

(2) Do competing land uses (gold mining, biofuel concession and extraction of natural resources by communities) and landscape features (altitude, slope, distance to rivers and roads) correlate with tree cover change between 2000 and 2010?

## Results

### Participatory rural appraisal

The community use areas (CUAs) around 31 communities adjacent to Hukaung Valley Wildlife Sanctuary (notified in 2004) were mapped, representing 49% of the 63 communities in the Hukaung Valley landscape. The 31 communities had been founded between seven and more than 200 years before the start of the study, with a mean community age of 50 years. In total, these CUAs supported an estimated 24,650 people, with a mean community size of 747 individuals (ranging from 40 to 3773 individuals). These 31 communities had an average CUA of 141.7 ± 102.9 (SD) km^2^, but this ranged from 13.4 km^2^ to 443.2 km^2^. The agricultural concessions overlapped with eight mapped CUAs, completely encompassing the CUAs of two communities, which housed around 1100 people. This meant that 15% of the total mapped CUA was inside agricultural concessions. Gold mines were found inside the CUAs of three communities, and two of these communities also overlapped with the Yazana concession. 230 local natural resources were considered important by at least one of the 1061 participants in 56 focus groups. Over 20% of the 1061 participants in the focus groups valued the bamboo wanet (*Dendrocalamus longispathus*), which is used mostly for walls and floors in house construction (Table 1). Another important resource, sambar deer (*Rusa unicolor*), was eaten by participants, but also sold to workers at the gold mines and in the concessions. Most of the ten most important resources (Table 1) are used for house construction and building. Participants may have ranked species as important by their frequency of use, but most of the ten important species were used in household construction, which is an occasional but ongoing activity, as households need to be reroofed every 2–5 years and much of the floor and walls are also regularly replaced. Population reductions in sambar deer and sagawa (*Machelia champaca*, an evergreen tree) were reported by all groups which discussed their population trends (21 and 26 groups respectively). Discussions of population changes in the other eight most important natural resources were more varied, with some groups reporting little change, while others reported increases or decreases. Two communities reported the bamboo waboe (*Dendrocalamus hookeri*) to be locally extinct. During focus groups it was stated that decreases of natural resources were due to increased demand for charcoal, meat, and house construction materials on migrant workers who came to work in the gold mines and concessions.

Quantitative information on income was not available at the household level, but there was evidence that communities adopted new economic opportunities; ten communities identified gold mining, panning and selling as important income sources, and residents of 11 communities were employed in agricultural concessions. However, income generating activities connected to natural resource extraction and the agricultural concessions were mostly conducted by households perceived by participants as lower income. For example, gold panning and selling of minor forest products was only carried out by lower income households. These households were also more often associated with hunting, rattan and bamboo collection and wage labour for agricultural concessions. In contrast, growing opium and gold dealing were only conducted by households perceived by participants as higher income.

### Tree cover change

#### Accuracy

**Figure 1:**
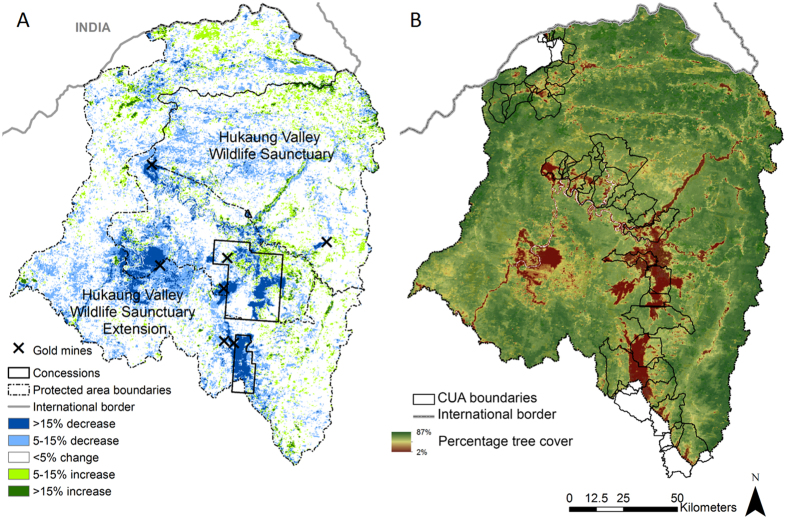
Tree cover and tree cover change in Hukaung Valley, Northern Myanmar. (**A**) Decreases in percentage tree cover in the Hukaung Valley landscape between 2000 and 2010 are high close to gold mines and inside the southern Yuzana biofuel concession. (**B**) Percentage tree cover in the Hukaung Valley landscape in 2010 and location of mapped community use areas (CUAs). Figures generated in ArcGIS from MODIS VCF data layers at 230 m resolution^47^, as described in the methods section.

Percentage tree cover across the Hukaung Valley landscape was spatially heterogeneous in 2000, and averaged 76.77 ± 10.13SD% as measured by the MODIS VCF product^47^. In forested areas (MODIS VCF > 25%, and classified as forest by a Landsat land classification), this decreased by a mean of 1.73% (−1.73, 95% CI: −1.75 to −1.70%) between 2000 and 2005, and an additional 0.59% (−0.59, 95% CI: −0.61 to −0.56%) between 2005 and 2010 (Fig. 1A). Between 2000 and 2005 however, only distance to gold mines was consistently correlated with changes in percentage tree cover (p < 0.05 in 90.5% of 200 models, <20% for all other variables). As distance to gold mines increased from 1 to 1000 m, mean percentage tree cover change increased by 0.58% (95% CI: 0.55–0.61%, back-transformed from average model estimates over 200 models). A decrease in the variance of the residuals of the model was found at higher altitudes between 2000–2005 and 2005–2010. There was also a positive relationship between altitude and changes in percentage tree cover in both time periods, meaning that greater tree cover losses were observed at lower altitudes (Table 2). Between 2005 and 2010, percentage tree cover change between 283 m and 1041 m (1^st^ and 3^rd^ quantile of altitude respectively) increased by 2.12% (95% CI 2.03–2.20, back-transformed from average model estimates over 200 models). Although this relationship was statistically significant in 84.5% of models between 2005–2010, it was only statistically significant for 16.5% of models between 2000–2005. Between 2005 and 2010, the two agricultural concessions granted in 2006 were strongly associated with changes in percentage tree cover, with 6.49% greater decreases in tree cover (−6.49, 95% CI: −6.22 to −6.76%, p < 0.05 in 97% of 200 models) within agricultural concessions when compared to outside (Fig. 1A).

**Figure 2:**
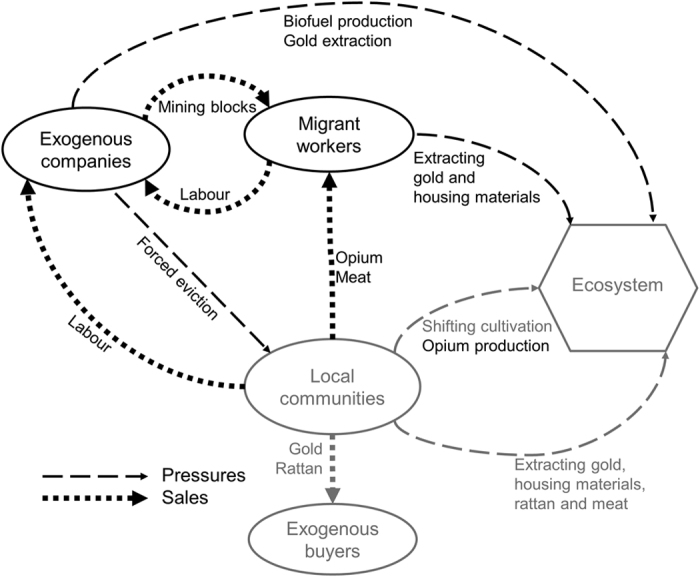
Changes in the socio-economic system and interactions with the ecosystem associated with industrial-scale extraction of natural resources in the Hukaung Valley Landscape. Interactions in the modified socio-economic system associated with these changes are shown in black, interactions present before 2000 are shown in grey.

Forested areas in 2000 in most of the Hukaung Valley landscape retained substantial tree cover in 2010 (mean VCF 75.76% ± 10.89SD, Fig. 1B), but percentage tree cover was extremely low inside some mapped CUAs, dropping from a mean of 74.95% (range: 25–86%) across all CUAs in 2000 to 69.98% (range: 4–86%) by 2010. Within CUAs, gold mines were the only factor associated with changes in tree cover between 2000 and 2005 (Table 3), with greater tree cover decreases in CUAs closer to gold mines. In contrast, between 2005 and 2010, CUAs inside agricultural concessions experienced 12% greater losses in percentage tree cover than CUAs outside concessions. We synthetized the information from the focus discussion groups, model results and reviewed literature as a conceptual framework to illustrate the dynamics Hukaung Valley Landscape (Fig. 2).

## Discussion

Although mining has been present within the Hukaung Valley landscape for decades, we found evidence of a spatial association between gold mines and tree cover loss. This is consistent with studies in South America, Africa and other parts of Asia where artisan and industrial gold mining is associated with deforestation^7^,^48^,^49^,^50^. For example, Paull *et al*.^49^ found a six-fold increase in forest loss in areas affected by the Freeport gold mine in Indonesia. Community reports (from the focus groups) that gold mines and agricultural concessions were responsible for local environmental degradation were consistent with the analysis of tree cover change between 2000 and 2010. From the data available, we are unable to comment on the impact of mining on forest cover in Hukaung before 2000, but there is evidence for a spatial association between tree cover decreases, gold mining and agricultural concessions between 2000 and 2010, and little evidence for greater decreases closer to local communities. Although opium cultivation by local households has been suggested as a significant driver of forest loss along river banks in the Hukaung Valley landscape^45^, no correlation was found in this analysis between distance to rivers and streams and forest degradation. As mining has been present within the Hukaung Valley landscape for decades before the period covered in this study, deforestation around mines may have occurred prior to our study. We cannot quantify if deforestation as a result of gold mining occurred before our study, but as we excluded all areas without substantial forest cover (<25% tree cover) in 2000, we can demonstrate the spatial relationship between gold mining and deforestation between 2000 and 2005.

Another impact of increasing industrial-scale land use in the Hukaung Valley are the new livelihood opportunities for local households that seek work and trading opportunities (Fig. 2). The changes in the relationships between local communities, gold mining and the creation of agricultural concessions in 2006 are complex, but appear to have greatest linkages with households perceived as the poorest in local communities. Zarin *et al*.^51^ describe forests as potential ‘poverty traps’, where forest dependence is associated with fewer income generating activates, resulting in a range of negative social and economic outcomes. An individuals’ ability to adopt new livelihood strategies is typically linked to social-economic status, including wealth, education and social capital^52^. Those with limited adaptive capacity may remain dependent on degraded forest resources. For example, higher levels of poverty are found among communities in forested areas of East-Kalimantan (Indonesia) compared to those in more deforested areas^53^. Communities within East-Kalimantan perceive proximity to forests as closely associated with poverty. In the Hukaung Valley landscape, it appears that poorer households are most likely to gain income from interactions with gold mining and agricultural concessions, but also show greater reliance on forest products and may therefore experience greater costs from negative feedbacks caused by forest degradation^54^. As these data show correlation rather than causation, it is impossible to state whether these households are perceived as poor because they engage in these activities or vice versa. Nevertheless, these results suggest caution when proposing industrial-scale resource extraction in new areas. Although these new industries may provide additional economic opportunities for local communities, these opportunities may not be equitably distributed^30^ and may further disadvantage rural poor who rely on natural resources and have limited adaptive capacity.

A previous study has provided an estimate of forest cover (75.45%) in 2000 and an annual estimate of 0.094% forest cover loss between 2000 and 2005 for Hukaung Valley Wildlife Sanctuary based on MODIS VCF and Landsat images^55^. One challenge to the robustness of the conclusions about tree cover change in our analysis is the lack of groundtruthing for tree cover loss. Although we validated the 2000 MODIS VCF product using a groundtruthed Landsat image, it was not possible to groundtruth the changes in forest cover between 2000 and 2010. The estimates of this prior study are based on a slightly different geographic area (this study uses current boundaries of Hukaung Valley Wildlife Sanctuary and thus a substantial expansion to the south-west) and methodology (notably, the calculation of forest change does not exclude non-forested areas), but provide a similar starting estimate and consistent direction of change as found in this study. Unlike the previous study, which found a positive relationship between forest cover loss and highways across Myanmar^55^, in our study there was no consistent significant relationship between distance to roads and forest cover loss. Roads are often used as a proxy for accessibility, and suitable agricultural lands at lower elevations are also assumed to be more easily accessed. The higher forest cover loss observed at lower altitudes in this study was congruous with other studies in Myanmar and elsewhere that find greater levels of deforestation at lower altitudes^56^,^57^,^58^,^59^,^60^.

One drawback of using satellite data of percentage tree cover as an indicator of environmental impacts of land uses is that it does not consider other impacts such as mercury poisoning through the gold extraction process, overhunting and fishing or overuse of specific species. For example, multiple pathways link mining activities and the degradation of ecosystems that support livelihoods in Western Ghana^48^, but not all of these pathways are associated with habitat cover. Although our analyses of the Hukaung Valley landscape did not find consistent evidence of greater tree cover losses close to communities, participants reported decreases in key natural resources, particularly sambar deer and housing materials. Local communities stated large populations of migrant workers in the concessions and gold mines were responsible for a reduction in staple resources, due to an increased demand for charcoal, meat, and house construction materials. Some migrant workers likely collect these resources for personal use, and households in local communities also collect and sell these resources to the migrants. Care must be taken when using local ecological knowledge as a source of information in species populations; shifting baselines, imprecise observation, and normative factors can all influence the reliability of information^61^,^62^. However, these reports are consistent with studies on the role of migration in frontier expansion and subsequent impact on ecosystems in other parts of the world^63^,^64^. This study did not explicitly explore the impacts of declining species populations on communities or ecosystems, but removal of vegetation such as the bamboo species *Dendrocalamus longispathus* may reduce the availability of grazing fodder for wild species. Similarly, declines in Sambar deer (*Rusa unicolor*) population may impact threatened predator species such as the Indochinese tiger (*Panthera tigris corbetti*), which are found within the Hukaung Valley Wildlife Sanctuary^65^,^66^. The Yuzana concession employs a total of 3,000 migrant workers, representing a substantial increase on the 5,000 individuals previously living in local communities within the concession area^67^. These local increases in demand for key resources are likely to be found throughout Myanmar as individuals migrate to new areas in search of economic opportunities. One potential solution, which could address high demand for particular plant species, are managed populations within CUAs. As new economic activities are planned, local communities could cultivate species used for house construction, and sell these to migrants as they arrive. Managed populations are one possible alternative to wild harvesting (e.g. as suggested for collared peccaries^68^ and medical plants^69^ in other systems). Cultivation of species currently harvested from the wild in the Hukaung Valley landscape and in other places in Myanmar might increase income for existing communities and reduce the pressures on the local environment by migrants. Some communities in the Hukaung Valley already have plantations of important wild plant species. Facilitating this approach across other communities in the Hukaung Valley and Myanmar may increase their resilience to further changes in the wider landscape.

Managed populations are consistent with existing governance structures in Myanmar. Governmental recognition of community managed forests in Myanmar is formalized through certified Forest User Groups that manage their own community area and activities such as reforestation. Collection of natural resources is permitted, but private agricultural use is not. The efficiency and inclusiveness of Forest User Groups varies^70^ but there is strong evidence that that some individuals gain diverse benefits from resources extracted in CUAs^71^. In Hukaung Valley, agricultural and gold mining companies have restricted community access to natural resources, both through destruction of forest within CUAs and the eviction of some communities and households^67^. The companies involved have breached several national forestry laws and the evictions were contested in a letter from communities within Hukaung Valley to the President of Myanmar (RT, personal observation). Our results show that there is significant overlap between agricultural concession areas and traditionally used CUAs. Simultaneously, many community members are employed in the gold mines and agricultural concessions. This highlights important links between communities and extractive industries in the Hukaung Valley. As such, further conflict between these groups could be detrimental to both sides. Adopting the Forest User Groups model, which seeks to manage resources at a landscape level, may reduce the risk of future land rights conflicts across Myanmar^72^.

Rapid changes in social and environmental conditions will likely occur across Myanmar in the near future as annual rates for economic growth are expected to be as high as 8% and tightly linked to natural resource exploitation^4^. This creates new challenges for the people and biodiversity of Myanmar, particularly among those groups that have limited capacity to adapt to and benefit from this change. This could be addressed through policy and institutional reform and the integration of environmental safeguards into economic development planning^73^. Conservation and development planning in Myanmar needs to account for the complex feedbacks between local communities and extractive industries in space and time. It also needs to take into account the effects of internal migration on ecosystems and natural resource use following expansion of new employment opportunities. Adopting a landscape approach that incorporates multiple scales of management, anticipates likely impacts and identifies underlying social-economic systems, may reduce negative feedbacks for both biodiversity conservation and local communities. In the context of Hukaung Valley, and other parts of Myanmar, this might be achieved through the development of Forest User Groups. These findings may be of particular interest to the Ministry of Environmental Conservation and Forestry in the government of Myanmar who administer Forest User Groups, local NGO’s and intergovernmental organisations and companies planning industrial expansion in Myanmar. Recent sociopolitical changes in Myanmar offer substantial opportunities for its citizens and biodiversity conservation^1^, but work is still required to safeguard against potential negative impacts of exogenous investments and integration into global markets^2^,^4^,^74^.

## Materials and Methods

### Study location

The Hukaung Valley landscape (26º23′N, 96º26′E) includes tropical and temperate broadleaf forests, conifer forests and some areas cleared for human activities including shifting cultivation^75^, as well as the recently created Hukaung Valley Wildlife Sanctuary (notified in 2004) and the Hukaung Valley Wildlife Sanctuary Extension (notified in 2010). Covering around 17,000 km^2^, these protected areas are smaller than the proposed area due to removal of a large central area (Fig. 1), but remain the largest terrestrial protected area in Southeast Asia and an area with land rights conflicts and a variety of economic activities^76^. In this paper, we consider a community to be a collection of households that are geographically associated and referred to using a collective name. Nevertheless, in all communities, local households use and manage the natural resources around their communities without being legally recognized as stakeholders in the local landscape. We use the term “community use area” (CUA) to describe areas used by a community and recognized as part of their landscape by the community itself and neighboring communities, regardless of whether this land is legally recognized as such.

### Mapping community use areas

CUAs of 31 communities within the Hukaung Valley landscape were mapped between 2005 and 2010 through collaborations between community inhabitants, the Nature and Wildlife Conservation Division (NWCD) of the Forestry Department of the Ministry of Environmental Conservation and Forests and the Wildlife Conservation Society (WCS) Myanmar field team. Communities adjacent to the Hukaung Valley Wildlife Sanctuary (notified in 2004) were selected for participation. The CUAs were mapped as part of participatory rapid rural appraisals conducted by NCWD and WCS Myanmar to initiate long-term relationships with communities and develop sustainable natural resource management plans. The NCWD and WCS field team met with the head of each community and explained their intention to map the areas used for crops, timber and non-timber forest products. Community elders and those considered knowledgeable were consulted about the traditional CUA boundaries and constructed a sketch map. Knowledgeable individuals in each community accompanied the NCWD and WCS team to map the sketched boundaries with a GPS. Boundaries between neighboring communities were established through joint meetings with both communities. Once the CUA was mapped and approved by community members, a shapefile of all CUAs was produced by WCS Myanmar (shown in Fig. 1B).

### Understanding valued local resources and interactions

In 28 communities (27 where the CUA was mapped, plus one additional community where the CUA was not mapped), male and female focus groups were formed (total 56 focus groups) and participants asked to list anything eaten, sold or extracted from forested areas surrounding the community. Focus groups are routinely used in the social sciences as a cost-effective method of gathering data on specific themes. They can be used to generate a broad range of data, including highlighting consistent and contrasting attitudes, perceptions and beliefs^62^. Responses in focus groups tend to be more reflective of social norms than those elicited during individual interviews and the role of vocal or influential participants should be considered when analysing the results. Male and female focus groups were run separately to encourage participation and freer expression by female participants. Multiple experienced facilitators were present in all focus groups. Each individual was given three counters and asked to place a single counter on the three resources most important for their livelihoods and family. Relative importance across all communities was calculated for all resources receiving at least one vote during this exercise. To control for variation in number of male participants (range 6–50, mean = 21) and female participants (range 6–59, mean = 17) in each community, and the variation in number of participants between communities, the proportion of male and female individuals voting for each resource was calculated for each community. An overall value was gained by taking the mean proportion across all communities for males and females. Subsequently, groups were asked to classify community households as high, middle and low income, giving livelihood activities associated with each group of households. 1061 individuals (of an estimated population of *ca*. 20,000) participated and provided information on the uses and management of key resources, and details of conflicts and issues associated with natural resource use. As part of the participatory rapid rural appraisals, the NCWD and WCS also collected information on the date communities were founded and number of residents.

### Analysis of changes in forest cover

We conducted separate analyses for the periods 2000–2005 and 2005–2010 to examine tree cover changes after two agricultural concessions were granted in 2006. We used the Moderate Resolution Imaging Spectroradiometer (MODIS) vegetation continuous fields (VCF) product for the years 2000, 2005, and 2010^47^, to calculate change in percent tree cover using the overall change in percentage tree cover between two time periods. The MODIS VCF product measures percentage tree cover in each 230 m resolution grid cell. Change in percentage tree cover between 2000 and 2010 are shown in Fig. 1A and generated using these data. Figure 1B is generated from VCF cover in 2010. Altitude and slope were calculated from the 30 m resolution ASTER-GDEM data product (made available by METI, Japan and NASA, USA) using the ‘raster’ package in R 3.1.2^77^ to create rasters with 230 m^2^ grid cells consistent with the MODIS VCF products. The VCF 2000 product was cross-validated using a ground-truthed supervised land cover classification of Landsat 7ETM+ imagery from February 2000–2001^78^ which covered the entire area of this study. The Landsat land cover classification had 15 classes, including four forest classifications: hill forest, secondary forest, closed evergreen forest and open evergreen forest. Across all classes the Landsat land cover image had an average of 91% accuracy, rising to 94% and 95% for the two most common forest types, hill forest and closed evergreen forest respectively (occupying 76% of the total area within the Hukaung Valley landscape in 2000–2001)^78^. To ensure that all locations included in the analysis were covered in forest in 2000, only locations within Hukaung Valley landscape which were classified as forest by the Landsat land cover image and greater than 25% tree cover in the 2000 MODIS image^79^ were considered forested and considered for inclusion in the analysis. 25% tree cover was chosen as a threshold to maintain consistency with other analyses of forest cover from MODIS VCF data^79^. Cross referencing this with the Landsat land classification image also excluded areas covered by bamboo or agriculture even if they had greater than 25% tree cover. In Kachin State, MODIS VCF 2005 data is highly correlated (R^2^ = 0.9708) with percent tree cover calculated from a Landsat 5 image^80^. Observed decreases in percentage tree cover in the Hukaung Valley landscape cannot be securely treated as estimates of loss of native primary forest, as neither the MODIS VCF nor the Landsat land cover type classification distinguishes between native and non-native forest types, or primary and secondary forest. Nevertheless, as there are no extensive areas of non-native forest in the Hukaung Valley landscape and the Landsat classification used for cross-validation excluded bamboo and shifting cultivation areas from the analyses, all losses of tree cover in this analysis are likely to be losses of primary or secondary (though not necessarily native) tree vegetation. Tree cover is of high importance for the Hukaung Valley landscape, and regardless of whether primary or secondary forest, forested areas can support certain animal populations, prevent soil erosion, ensure water cycling and sequester carbon^81^,^82^. In addition to local community use, gold extraction and commercial agricultural production, we also included altitude, slope and distance to rivers as geophysical variables that could be correlated with tree cover change. As rivers are used by local people for access, any spatial correlation with changes in tree cover could be due to either human activities or ecological processes. Access is an important driver of environmental degradation^11^,^55^,^83^, so we included distance to trails and roads as a potential factor impacting tree cover. Within CUAs, we also investigated the role of population density and the number of years since the community was founded on tree cover change. Shapefiles of roads, rivers, communities and gold mines were digitalised from Earth imagery (LANDSAT/SPOT) and ground-truthed by the WCS Myanmar team using GPS. Identically sized rasters of the distance in meters to roads, rivers, communities and major gold mines were created. A raster where each cell was classified as either being inside or outside an agricultural concession was also developed. During the period of study there were also short lived rattan and bamboo concessions and several small sawmills in the Hukaung Valley landscape. The locations of these have not been mapped so it was not possible to include them in the model, but they are likely to contribute to forest degradation and loss in the surrounding areas.

### Modelling

To investigate the impact of different human activities on forest across the whole landscape, analyses were conducted on the 230 m^2^ raster grid squares which were in forested areas of the Hukaung Valley landscape in 2000 (including inside CUAs). For each grid square, dependent and independent variables were extracted from the rasters using the package ‘raster’ in R 3.2.1^77^. To normalize their distribution, slope and independent variables that were measured as distance, and the variable slope, were square root transformed, and the log of altitude was used. In addition to normalizing residuals, the square root of distances to features more closely approximates anticipated relationships with forest cover loss, where effects are strongest closest to the feature and decrease non-linearly over increasing distances. We used generalized least squares models in the R statistical environment version 3.2.1 with change in tree cover in two periods (2000–2005 and 2005–2010) as the dependent variables. Tree cover at the end of the period (2005 and 2010 respectively) was subtracted from tree cover at the start of the period (2000 and 2005 respectively). A negative sign for the dependent variable shows that tree cover has decreased over the period, and is positive if tree cover has increased. The dataset comprised of 359641 observations. Due to the exponentially increasing computer power needed with sample size in GLS model with spatial autocorrelation structures, we had to restrict the number of observations evaluated. We limited ourselves to a sample size of 2000 observations. To account for the uncertainty associated to using only a subset of the observations, we adopted a bootstrapping approach in which 200 models were fitted to 200 randomly selected samples of the global dataset. Then the estimates of model coefficients were extracted to build credible intervals and the p-values of each model were obtained. We used this p-values to estimate the proportion of models that were statistically significant. Each dependent variable was obtained after deducting the original forest cover to the final forest cover of the time period studied. We checked for potential problems of multicollinearity with generalized variance inflation factors, but all were less than four^84^. We plotted the residuals of the models versus fitted values and the residuals versus each explanatory variable of change in forest cover to assess problems of heteroscedasticity. The variance of model residuals strongly decreased at greater distances from water features, so we compared fixed, power, exponential and constant power variance structures for the squareroot of distance to water features to control for this. Semivariogram plots of the residuals indicated problems of spatial correlation. To correct for this, we compared models with different spatial autocorrelation structures using AIC. We found that the rational quadratic covariance function was able to correct for spatial correlation for both time periods.

To access the impact of different drivers of forest change within community use areas, we generated 40 random points inside each mapped CUA within the Hukaung Valley landscape. Additional independent variables (population density in the CUA and number of years before 2010 that the community was founded) were extracted from the participatory rapid rural appraisals at each community. For consistency in the analysis, two communities were excluded as the majority of their CUA lay outside the Hukaung Valley landscape, leaving a total of 29 communities. For each community, the mean distance to gold mines, accessibility and water bodies were calculated from the 40 random locations, and each community was classified as either inside (8 communities) or outside (21 communities) an agricultural concession. For the eight communities classified as inside an agricultural concession, between 2% and 100% of the CUA lay inside the concession (median = 54%). As there were only 29 communities, altitude and slope could not be considered in this analysis, due to insufficient degrees of freedom for robust analyses. Altitude and slope were excluded as an exploratory analysis did not suggest any relationship between mean altitude or slope and changes in tree cover within CUAs.

## Additional Information

**How to cite this article**: Papworth, S. *et al*. The impact of gold mining and agricultural concessions on the tree cover and local communities in northern Myanmar. *Sci. Rep.* **7**, 46594; doi: 10.1038/srep46594 (2017).

**Publisher's note:** Springer Nature remains neutral with regard to jurisdictional claims in published maps and institutional affiliations.

## Figures and Tables

**Table 1:** The natural resources most valued by local communities in Hukaung Valley Sanctuary.

Rank	Local name	Scientific name	Resource type	Females naming resource as important (%)	Males naming resource as important (%)	Uses
1	Wanet	*Dendrocalamus longispathus*	Bamboo	25.2	21.9	House construction, rafts, shoots are eaten
2	Sagawa	*Machelia champaca*	Evergreen tree	20.6	25.5	Household construction
3	Yone	*Salacca secuda*	Palm	20.1	15.3	House construction and materials
4	Tawhtan	*Livistonia jenkinsiana*	Palm	15.7	18.2	House construction and materials
5	Laywar	Unidentified bamboo	Bamboo	13.1	8.4	Baskets and other woven handicrafts
6	Khalaung	*Dysoxylum binectariferum*	Evergreen tree	10.9	7.9	House construction and materials
7	Sat	*Rusa unicolor* (Sambar deer)	Animal	11.5	4.9	Crop pest hunted for meat and sold to migrant workers
8	Shwe	Not applicable	Gold	7.8	6.7	Sold
9	Thet kei	Unidentified grass	Grass	7.9	5.8	House construction
10	Waboe	*Dendrocalamus hookeri*	Bamboo	7.7	5.5	Fencing, house construction, rafts

**Table 2:** Factors associated with changes in percentage tree cover (means with 95% confidence intervals in square brackets) within the Hukaung Valley landscape during two time periods.

Factor (transformation)	Change in percentage tree cover 2000–2005	Change in percentage tree cover 2005–2010
Altitude (metres, log)	0.475 [0.400, 0.550] (16.5%)	1.625 [1.560, 1.689] (84.5%)
Slope (degrees, square root)	−0.018 [−0.043, 0.008] (6.0%)	−0.09 [−0.118, −0.063] (9.5%)
Distance to drainage (rivers and streams, metres, square root)	0.005 [0.004, 0.007] (5.5%)	0.004 [0.002, 0.006] (4.5%)
Distance to roads (metres, square root)	−0.008 [−0.009, −0.008] (25.0%)	−0.001 [−0.002, −0.000] (0.5%)
Distance to communities (metres, square root)	0.008 [0.007, 0.008] (10.5%)	0.009 [0.008, 0.010] (11.5%)
Inside concession (binary: yes/no)	−0.441 [−0.634, −0.248] (2.5%)	−6.489 [−6.220, −6.758] (97.0%)
Distance to gold mine (metres, square root)	0.019 [0.018, 0.020] (90.5%)	0.007 [0.006, 0.007] (1.5%)

Distances are measured in metres. Generalized least squares with constant power of covariance structure for altitude and a rational covariance function to correct for spatial correlation, over 200 repeated runs. Overall model p values were under 0.05 for 80% of models for 2000–2005, and 100% models for 2005–2010. Figures in square brackets show 95% CI of the means for 200 runs. P-values are summarised for each factor in round brackets as the percentage of values across 200 runs less than 0.05.

**Table 3:** Factors associated with changes in percentage tree cover within 29 community use areas (CUAs) in Hukaung Valley Sanctuary during two periods: 2000–2005 and 2005–2010.

Factor (unit)	Mean change in percentage tree cover 2000–2005	Mean change in percentage tree cover 2005–2010
Distance to water bodies (km)	−0.05 ± 1.27	−1.56 ± 2.54
Distance to roads (km)	0.44 ± 0.53	1.07 ± 1.07
Distance to gold mines (km)	**0.18** ± **0.04**	0.04 ± 0.08
Concession (categorical: inside/outside)	2.70 ± 1.34	**−12.29** ± **2.68**
Population density (people per km^2^)	0.03 ± 0.08	−0.11 ± 0.17
Time since settled (years)	−0.04 ± 0.02	0.06 ± 0.04
Adjusted R^2^	0.53	0.47

Generalized linear model, estimate and standard error, variables with p values below 0.05 are shown in bold.
